# Antileishmanial effects of γCdcPLI, a phospholipase A_2_ inhibitor from *Crotalus durissus collilineatus* snake serum, on *Leishmania* (*Leishmania*) *amazonensis*

**DOI:** 10.1590/0074-02760220225

**Published:** 2023-11-27

**Authors:** Marina Neves Gonçalves, Daiana Silva Lopes, Samuel Cota Teixeira, Thaise Lara Teixeira, Vitor de Freitas, Tássia Rafaella Costa, Sarah Natalie Cirilo Gimenes, Isabella Mitie de Camargo, Guilherme de Souza, Marcelo Santos da Silva, Fernanda Van Petten de Vasconcelos Azevedo, Kathleen Fernandes Grego, Luísa Carregosa Santos, Vinícius Queiroz Oliveira, Claudio Vieira da Silva, Renata Santos Rodrigues, Kelly Aparecida Geraldo Yoneyama, Patricia Bianca Clissa, Veridiana de Melo Rodrigues

**Affiliations:** 1 Universidade Federal de Uberlândia, Instituto de Biotecnologia, Laboratório de Bioquímica e Toxinas Animais, Uberlândia, MG, Brasil; 2 Universidade Federal da Bahia, Instituto de Biociências, Vitória da Conquista, BA, Brasil; 3 Universidade Federal de Uberlândia, Instituto de Ciências Biomédicas, Departamento de Imunologia, Uberlândia, MG, Brasil; 4 Universidade Federal de São Paulo, Escola Paulista de Medicina, Departamento de Microbiologia, Imunologia e Parasitologia, São Paulo, SP, Brasil; 5 Instituto Butantan, Laboratório de Imunopatologia, São Paulo, SP, Brasil; 6 Universidade de São Paulo, Instituto de Química, Departamento de Bioquímica, São Paulo, SP, Brasil; 7 Instituto Butantan, Laboratório de Herpetologia, São Paulo, SP, Brasil

**Keywords:** snake serum, Crotalus durissus collilineatus, phospholipase A_2_ inhibitors, γCdcPLI, leishmaniasis treatment

## Abstract

**BACKGROUND:**

Leishmaniasis, a neglected disease caused by the parasite *Leishmania*, is treated with drugs associated with high toxicity and limited efficacy, in addition to constant reports of the emergence of resistant parasites. In this context, snake serums emerge as good candidates since they are natural sources with the potential to yield novel drugs.

**OBJECTIVES:**

We aimed to show the antileishmanial effects of γCdcPLI, a phospholipase A_2_ inhibitor from *Crotalus durissus collilineatus* snake serum, against *Leishmania* (*Leishmania*) *amazonensis*.

**METHODS:**

Promastigotes forms were exposed to γCdcPLI, and we assessed the parasite viability and cell cycle, as well as invasion and proliferation assays.

**FINDINGS:**

Despite the low cytotoxicity effect on macrophages, our data indicate that γCdcPLI has a direct effect on parasites promoting an arrest in the G1 phase and reduction in the G2/M phase at the highest dose tested. Moreover, this PLA_2_ inhibitor reduced the parasite infectivity when promastigotes were pre-treated. Also, we demonstrated that the γCdcPLI treatment modulated the host cell environment impairing early and late steps of the parasitism.

**MAIN CONCLUSIONS:**

γCdcPLI is an interesting tool for the discovery of new essential targets on the parasite, as well as an alternative compound to improve the effectiveness of the leishmaniasis treatment.

Leishmaniasis is a set of complex and multifaceted syndromes, with different clinical manifestations, caused by protozoa of the species of the genus *Leishmania*.^1^ Although leishmaniasis has a wide distribution worldwide, most cases occur in the American, Asian, and African continents.^2^ According to the Pan American Health Organization (PAHOS), leishmaniasis affected 18 countries in America in 2019, with Brazil being the country with the most notifications (about 15,000 cases).^2^

In the Americas, leishmaniasis is associated with at least 15 *Leishmania* species, belonging to the subgenera *Viannia*, *Leishmania* and *Mundinia*, allocated into the subfamily Leishmaniinae.^3^ ^,^ ^4^ ^,^ ^5^ ^,^ ^6^ ^,^ ^7^ ^,^ ^8^ *Leishmania* can cause damage to the skin, mucosa, and visceral organs. The main forms are Cutaneous Leishmaniasis (CL), Diffuse Cutaneous Leishmaniasis (DCL), Mucocutaneous Leishmaniasis (MCL), Anergic Diffuse Cutaneous Leishmaniasis (ADCL) and Visceral Leishmaniasis (VL).^2^ ^,^ ^9^ The most common clinical form is CL, while VL is the most severe form and, in most cases, fatal if left untreated.^9^ ^,^ ^10^

CL is a widespread tropical infection caused by numerous different species of *Leishmania* that are transmitted by sandflies.^8^ Among them, one can highlight *Leishmania (Leishmania) amazonensis*, which affects skin, causing ulcers characteristic of cutaneous leishmaniasis.^11^ ^,^ ^12^ However, *L. (L.) amazonensis* has been also associated with a remarkably diverse clinical manifestations, such as DCL, ADCL and, less frequently, with MCL and VL.^13^ ^,^ ^14^ In patients with no known causes of immunodeficiency, CL may progress to an absence of specific cellular response (anergy) which characterises the rare diffuse cutaneous leishmaniasis (DLC).^11^

In Brazil, the anergic-multiparasitic end of the leishmaniasis spectrum is exclusively associated with infection caused by *L. (L.) amazonensis*. Evidences suggest that this species has a particular capacity to negatively interfere with several immunological mechanisms necessary for the generation of an effective immune response.^14^ Therefore, these peculiarities highlight the urgency of further studies addressing possible alternative treatments against *L.* (*L.*) *amazonensis*.^7^

Drug therapy for all forms of leishmaniasis aims at ensuring adherence to treatment, alleviating symptoms caused by the disease, safely administering indicated medications, controlling and/or minimising the occurrence of adverse effects.^15^ Currently, therapy against leishmaniasis is based on the use of drugs that are associated with serious deficiencies such as toxicity, prolonged administration and possible emergence of resistance by the parasite.^15^

The complexity of leishmaniasis symptoms is largely a result of the parasite’s virulence, which among several actions can induce responses in the host cell, such as changes in lipid metabolism that lead to plasma membrane remodelling by membrane phospholipid turnover, then modulating the process of adhesion and invasion of the parasite.^16^ In this context, studies have reported that the secretion of endogenous phospholipases present in protozoa such as *Trypanosoma cruzi* and *L. (L.) amazonensis* increases the ability of the parasites to infect host cells.^17^ ^,^ ^18^ ^,^ ^19^

Literature findings have demonstrated the role of some molecules with anti-phospholipase action on leishmaniasis. Borges et al.,^20^ using polyclonal antibodies with high avidity and affinity for specific antigenic toxin epitopes of phospholipases A_2_ (PLA_2_s) from snake venom, demonstrated that these antibodies possibly recognise PLA_2_s present in the *L. (L.) amazonensis*, compromising its ability to establish infection in the host. Similarly, Bordon and collaborators^21^ demonstrated a selective action of three phospholipase A_2_ (PLA_2_) inhibitors against *L. (L.) amazonensis*, which was illustrated by a reduction in the lesion size and skin parasitism in infected BALB/c mice.

Natural products have been extensively studied in regards its anti-PLA_2_ activity, especially compounds from plants extracts, marine organisms and snakes.^22^ Venomous and non-venomous snakes display PLA_2_ inhibitory proteins, named PLIs, which are serum globular proteins and possess the unique ability to neutralise the enzymatic and toxic components of snake venom PLA_2_s.^23^ ^,^ ^24^ ^,^ ^25^ PLIs are classified into types α, β and γ, according to structural features, based on common motifs found in other proteins with diverse physiological properties.^26^ γ-type inhibitors (γPLIs) possess the group with the highest number of subunits. The monomers with molecular masses around 20-31 kDa are typically establish non-covalent oligomers of three to six subunits, which form in response to temperature changes.^27^ Moreover, γPLIs contain two conserved cysteine-rich domains, termed three-finger protein domain (TFPD), which are suggested to play a role in PLA_2_ recognition.^28^

γCdcPLI, a γ-type PLA_2_ inhibitor isolated from *Crotalus durissus collilineatus* snake serum, has been explored for its therapeutic properties.^27^ ^,^ ^29^ ^,^ ^30^ It was firstly isolated and chemically characterised as an oligomeric protein with 23 kDa by monomer, capable of inhibiting PLA_2_-induced biological activities, such as oedema and myotoxicity.^27^ γCdcPLI showed interesting antitumor and antiangiogenic properties, which appear to be related to the modulation of the PI3K/AKT pathway.^29^ γCdcPLI inhibited gene expression of PI3KR1, Akt1 and Akt3. Moreover, the use of γCdcPLI decreased the active form of Akt (p-Akt) and the PGE2 level in MDA-MB-231 cellular supernatant, thus suggesting a possible interaction between this inhibitor and endogenous PLA_2_s.^29^

Since endogenous PLA_2_s can be involved in parasite virulence and maintenance in vertebrate hosts, they can be considered a possible target for studies that aim to better understand the parasite infection process that leads to leishmaniasis.^20^ ^,^ ^31^ ^,^ ^32^ ^,^ ^33^ ^,^ ^34^ Here, we presented for the first time the antiparasitic effects of γCdcPLI on the proliferation and infectivity of *L.* (*L.*) *amazonensis* promastigotes. Our findings may pave the way for further investigations on the pathogenesis of the parasitic disease, as well as for the development of new therapeutic approaches against leishmaniasis.

## MATERIALS AND METHODS

*Crotalus durissus collilineatus serum and Bothrops pauloensis crude venom* - The serum of *C. d. collilineatus* (Cdc) was obtained from specimens maintained in the Reptiles Sector of the Federal University of Uberlândia, Minas Gerais, Brazil. This serpentarium was registered in the Brazilian Institute of Environment and Renewable Natural Resources - IBAMA (nº 301286). The snake blood was collected periodically and the serum was obtained by centrifugation at 5,000 *g* for 10 min at 4ºC. The serum was stored at -20ºC. *B. pauloensis* crude venom was collected from snakes kept at the Ceta serpentarium, Animal Toxin Extraction Center, Ltda. - CNPJ: 08.972.260/0001-30, Morungaba, SP, Brazil. This serpentarium has undergone IBAMA registration and obtained authorisation for the use of renewable natural resources (nº 2087163).

*Isolation of PLA*_*2*_ *inhibitor (γCdcPLI) from Cdc serum* - γCdcPLI was purified from the same specimens of Cdc serum in two sequential chromatographic steps, as previously published by Gimenes et al.^27^ First, 98 mg of serum was dissolved into 1 mL 0.05 M sodium phosphate buffer (pH 6.5) containing 0.2 M NaCl and applied on a Q-Sepharose column (GE Healthcare - United Kingdom) previously equilibrated with the same buffer. Fractions (Q1 to Q5), eluted with 0.05 M sodium phosphate buffer (pH 6.5) with crescent concentrations of NaCl (0.2 M, 0.35 M, 0.5 M and 0.7 M) at a flow rate of 12 mL/h at room temperature, were monitored at Abs 280 nm (spectrophotometer Ultrospec 1000 UV/visible, Pharmacia Biotech - United States). Fractions were lyophilised and stored at -20ºC.

Q4 fraction (8.5 mg) with inhibitory activity on phospholipases A_2_ (data not shown) was further submitted to NHS Hitrap (N-hydroxysuccinimide) affinity column immobilised with PLA_2_ BnSP-7.^18^ Affinity column was equilibrated with 10 mM Tris-HCl buffer (pH 7.5) and the inhibitor was eluted with 100 mM glycine-HCl buffer (pH 2.0). Fractions of 1 mL/tube were collected at a flow rate of 0.1 mL/min using an AKTA prime plus (Amersham Biosciences - United Kingdom). pH of the eluted samples was immediately adjusted with 1 M Tris-HCl buffer (pH 8.0).

The protein concentration was determined using Bradford reagent (Sigma, B6916) according to Bradford (1976).^35^ The homogeneity of protein was assessed by 12.5% sodium dodecyl sulfate-polyacrylamide gel electrophoresis (SDS-PAGE).

*Inhibition of PLA*_*2*_ *activity* - The PLA_2_ inhibition assay was determined according to De Haas and Postema.^36^ Phospholipase activity was measured using egg yolk as a substrate in the presence of 0.03 M sodium deoxycholate and 0.6 M CaCl_2_. In order to evaluate the inhibitory effect of γCdcPLI on PLA_2_ activity, the γCdcPLI was incubated with *B. pauloensis* venom at different venom:γCdcPLI (m/m) ratios for 30 min at 37ºC. Results were in triplicate and expressed in mEqNaOH/mg/min.

*Cell culture and parasite maintenance* - Immortalised macrophages (macrophages C57) were derived from the bone marrow of C57BL/6 mice and maintained according to Araujo et al.^37^ *L. (L.) amazonensis* promastigotes (IFLA/BR/67/PH8) were maintained in Brain Heart Infusion - HiMedia medium supplemented with 10% foetal bovine serum (FBS) (Cultilab, Campinas, Brazil), 100 mg of gentamicin/mL and 2 mM L-glutamine (GibcoBRL, Life Technologies, New York) at 23ºC. Promastigotes in the stationary phase (metacyclic) were used in the experiments.

*Cellular viability in promastigote forms of L. (L.) amazonensis* - Cytotoxicity assays in the presence or absence of γCdcPLI were performed on promastigote forms by the 3-(4,5-dimethylthiazol-2-yl)-2,5-diphenyl tetrazolium bromide (MTT) assay, as previously described.^38^ ^,^ ^39^ ^,^ ^40^ Promastigotes (1.0 x 10^6^ parasites/well) were placed in 96-well culture plates and incubated in two-fold serial dilution of γCdcPLI (from 0.781 to 50 µg/mL) for 24 h at 23ºC. Control parasites were incubated with medium only. After 24 h, promastigotes were incubated with 5 mg/mL MTT for 3 h at 23ºC. Formazan crystals were dissolved by adding 100 μL of PBS (137 mM NaCl, 2.7 mM KCl, 10 mM Na_2_HPO_4_ and 2 mM KH_2_PO_4_) containing 10% SDS and 0.01 M HCl. After 18 h, the absorbance was measured in a multi-well scanning spectrophotometer (MultiskanGO, Thermo Scientific) at 570 nm. Results were expressed as the percentages of viable cells in relation to untreated control (100% viability). Dose response inhibition curves [Log (inhibitor) vs. normalised response - Variable slope] were calculated.

*Cellular viability in macrophages C57* - Cell viability in the presence or absence of γCdcPLI was evaluated in macrophages C57 by MTT assay. Briefly, cells were cultured in 96-well plates (3.0 x 10^4^ cells/200 µL/well) in a medium supplemented with FBS for 24 h at 37ºC and 5% CO_2_. Then, cells were treated in two-fold serial dilution of γCdcPLI (from 0.781 to 50 µg/mL) in RPMI 1640 medium. After 24 h, supernatants were discarded and 10 µL of MTT (5 mg/mL) and 90 µL of 10% FBS medium were added to each well for 3 h, under the same culture conditions. Subsequently, formazan crystals were dissolved by adding 100 μL of PBS containing 10% SDS and 0.01 M HCl and, after 18 h, the optical density was determined at 570 nm on a plate reader. Cell viability was reported in percentages (viability %), with the absorbance of cells incubated only with culture medium considered as 100% viable. Dose response inhibition curves [Log (inhibitor) vs. normalised response - Variable slope] were calculated. The selectivity index (SI) was determined as the ratio between the half-maximal cytotoxic concentration (CC_50_) for macrophages C57 and the half-maximal inhibitory concentration (IC_50_) against *L. (L.) amazonensis*.

*Inference of the cell cycle phases based on DNA content analysis* - The cell cycle phases were inferred based on the DNA content analysis, as previously described.^41^ ^,^ ^42^ ^,^ ^43^ ^,^ ^44^ Exponentially growing promastigotes of *L. (L.) amazonensis* (1.0 × 10^6^ cells/well) were plated in 96-well micro-plates and treated with γCdcPLI (10 and 50 μg/mL) or culture medium (control group) for 24 h at 23ºC. Then, parasites were harvested and fixed in 70% ethanol for 18 h at 4ºC. To ensure that only the DNA was stained, parasites were incubated with RNase A (100 μg/mL) and propidium iodide (PI) (10 μg/mL) for 45 min in the dark at 37ºC. Cell cycle was analysed by a FACS CantoII (BD), and the data were obtained using Flow Jo software (version 7.6.3).

*Invasion assays* - Invasion assays were carried out following a published study, with minor modifications.^45^ Macrophages C57 (5.0 x 10^5^ cells/well) were cultured in a 24-well plate containing 13-mm coverslips in each well. After adhesion, the cells were submitted to two distinct experimental models: (i) macrophages C57 were pre-treated or not with 10 and 50 µg/mL of γCdcPLI for 24 h, and then infected with *L. (L.) amazonensis* promastigotes, with a multiplicity of infection (MOI) of 10 parasites per 1 cell, for 4 h at 23ºC; (ii) Promastigote forms of *L. (L.) amazonensis* (with a MOI of 10:1) were pre-treated or not with 10 and 50 µg/mL of γCdcPLI for 1 h, and allowed to invade the cells for 4 h at 23ºC. For both protocols, cells were fixed with Bouin’s solution (HT10132 Sigma Aldrich) and stained with Giemsa (1:20 - P3288 Sigma Aldrich).^37^ Finally, the coverslips were analysed under a light microscope to assess the following parameters: number of cells with invaded parasites (invasion rate) and total number of parasites invaded to these cells in a total of 200 cells examined randomly. Three independent experiments were performed in triplicate for each treatment.

*Intracellular killing assay* - Macrophages C57 (2.0 x 10^5^ cells/well) were cultured in a 24-well plate containing 13-mm coverslips in each well, and infected with a MOI of 10:1 of promastigote forms of *L. (L.) amazonensis* for 4 h at 23ºC. Cells were carefully rinsed several times with PBS to remove the excess of extracellular parasites. Next, cells were incubated with twofold serial dilutions of γCdcPLI (ranging from 50 to 1.56 μg/mL), or culture medium for 24 h 37ºC and 5% CO_2_. Also, the present study used the amphotericin B (1 μg/mL) (Sigma #1397-89-3) as a gold standard drug against leishmaniasis.^46^ Finally, the cells were fixed and stained as mentioned above, and the total number of intracellular amastigotes were counted in a total of 100 infected cells examined randomly an optical microscope.

*Statistical analysis* - Data are expressed as mean ± standard deviation (SD) of experiments performed at least three times in triplicate. All data were first checked for normal distribution. Differences between two groups were determined by Student’s t test (two-tailed) and Mann-Whitney test for parametric or non-parametric data, respectively. Differences among multiple groups were assessed by one-way analysis of variance (ANOVA) test with Dunnett’s multiple comparisons test, for parametric data, or by Kruskal-Wallis test with Dunn’s multiple comparison post-test, in the case of non-parametric data (GraphPad Prism Software version 8.00). Data were considered statistically significant at p < 0.05.

## RESULTS AND DISCUSSION

**Fig. 1:**
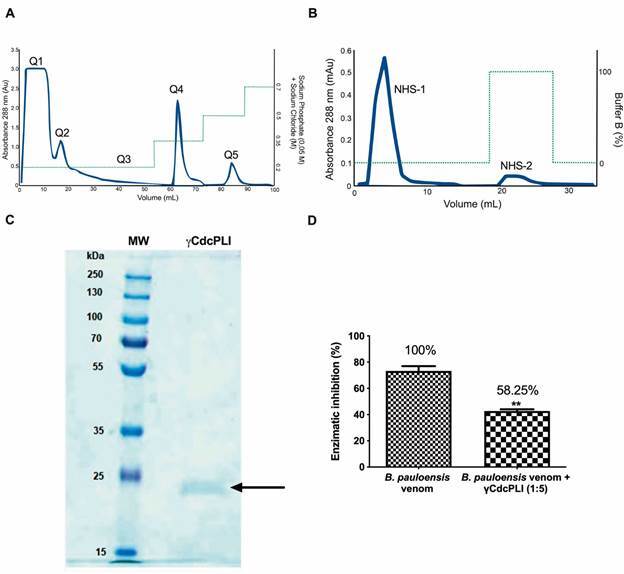
isolation of γCdcPLI from *Crotalus durissus collilineatus* snake serum. (A) 98 mg of lyophilised serum was dissolved in 1 mL of 0.05 M sodium phosphate buffer (pH 6.5) containing 0.2 M NaCl, applied to ion exchange chromatography (Q-Sepharose), equilibrated and eluted with buffer 0.05 M sodium phosphate with different NaCl concentrations (0.2, 0.35, 0.5 and 0.7 M) at a flow rate of 12 mL/h 25ºC. (B) NHS-Hitrap affinity chromatography (N- hydroxysuccinimide) coupled with BnSP-7 of Q4 fraction (8.7 mg) in (Buffer A: 10 mM Tris-HCl buffer, pH 7.5; Buffer B: 100 mM glycine-HCl buffer, pH 2.0). (C) 12.5% (w/v) sodium dodecyl sulfate-polyacrylamide gel electrophoresis (SDS-PAGE) of γCdcPLI: MW: molecular weight markers (250, 130, 100, 70, 55, 35, 25 and 15 kDa). The black arrow indicates the isolated protein. (D) Phospholipase A_2_ (PLA_2_) activity inhibition (*Bothrops pauloensis* venom: γCdcPLI, 1:5; w/w). NHS-2 fraction (0.5 mg) contains the γCdcPLI inhibitor, according to Gimenes et al.^27^. Data are expressed as mean ± standard deviation (SD). Significant differences were determined using Unpaired Student’s t test (two-tailed). Differences were considered significant when p < 0.05.

*Isolation of γCdcPLI from Cdc serum* - Here, we described the antileishmanial effects induced by γCdcPLI isolated from Cdc snake serum. Initially, we isolated γCdcPLI according to Gimenes et al.,^27^ with two chromatography steps by ion exchange chromatography on Q-Sepharose and by affinity on NHS Hitrap (N-hydroxysuccinimide) immobilised with the PLA_2_-BnSP-7 [Fig. 1 A, B and Supplementary data (Figure)]. Affinity chromatography has been employed by other authors to isolate different classes of inhibitors.^47^ The success of this method is related to the specificity and purity of the isolated proteins.^27^

The fractionation of Cdc serum on a Q-Sepharose Fast Flow column produced five major protein peaks, called Q1 to Q5 (Fig. 1A). The inhibitory effect of all fractions on PLA_2_ activity was tested (data not shown). The Q4 fraction, which showed PLA_2_ activity inhibition, was applied to an NHS Hitrap (N- hydroxysuccinimide) affinity column immobilised with PLA_2_-BnSP-7, resulting in two fractions, NHS-1 and NHS-2 (Fig. 1B). Similar to Gimenes et al.,^27^ NHS-2 fraction, which contains the γCdcPLI, was shown to be homogenous with a unique peak and a single band with a *Mr* of approximately 23kDa (Fig. 1C). This protein represented 0.5% of the Cdc serum and was able to inhibit 58.25% the PLA_2_ activity induced by *B. pauloensis* crude snake venom at the ratio 1:5 (*B. pauloensis* venom:γCdcPLI, m/m) (Fig. 1D). Gimenes et al.,^27^ showed the efficiency of the inhibitory γCdcPLI against acidic and basic Asp-49 PLA_2_s (BpPLA_2_-TXI and BthTX-II, respectively). The authors demonstrated that γCdcPLI can inhibit 100% of the activity of different types these PLA_2_. Furthermore, Gimenes et al.,^26^ determined the value of interaction between a PLA_2_ from honey bee and the recombinant form of γCdcPLI. The authors demonstrated a higher capacity of inhibitor to interact with the PLA_2_, which Kd value around 1.48 μM. Here, we observed a lower inhibition percentage of inhibitor against the whole snake venom. This finding suggests that due to snake venom is a rich mixture of a different types of toxins, among them different classes of PLA_2_,^48^ the protein interaction is affected, which contributes to a decrease in the capacity of the inhibitor to recognise, bind, and inhibit the enzymatic activity.^49^ This capacity of γPLIs in inhibiting PLA_2_ activities has been already described in the current literature. Oliveira et al.^30^ showed that the **γ**BjussuMIP isolated from *B. jararacussu* plasma was able to inhibit oedema, myotoxic, cytotoxic and bactericidal effects induced by bothropic PLA_2_s, which show the remarkable scenario for the use of these molecules as tools for the treatment of diseases.

**Fig. 2:**
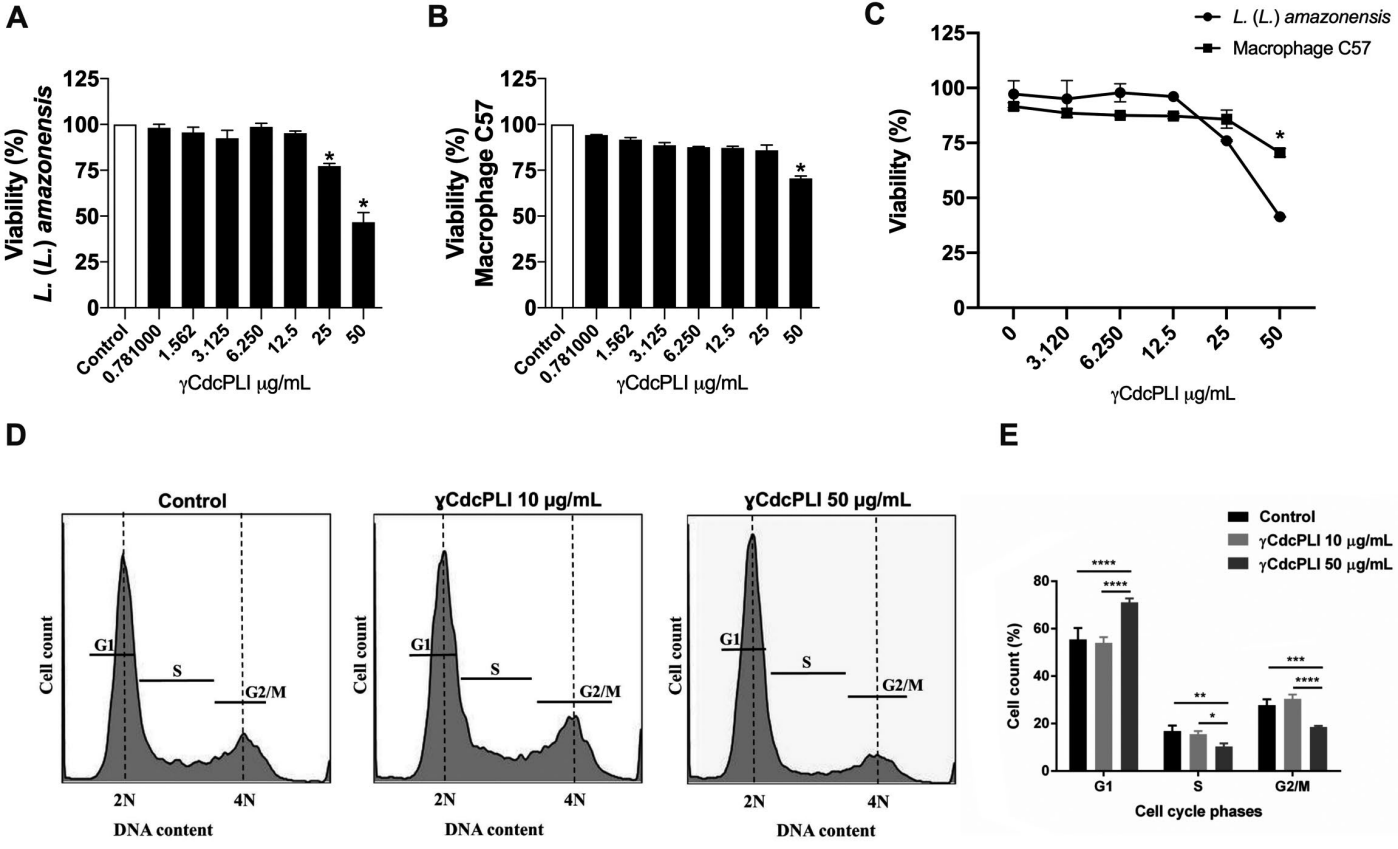
effects of γCdcPLI inhibitor on the viability and cell cycle progression. Viability assay of both (A) *Leishmania (Leishmania) amazonensis* promastigotes and (B) Macrophages C57 treated with γCdcPLI ranging from 0.781 to 50 µg/mL for 24 h. For the positive controls of viability, cells and parasites were treated with RPMI and LIT media only, respectively. (C) A comparative analysis of viability between the effects of γCdcPLI on cells and parasites. (D, E) Representative histograms and graph showed that γCdcPLI (50 µg/mL) promoted a significant cell cycle arrest at G1 phase at 24 h post-treatment. Data are expressed as mean ± standard deviation (SD). Significant differences were determined using one-way analysis of variance (ANOVA) and Dunnett’s multiple comparisons test. Differences were considered significant when p < 0.05.

*γCdcPLI decreases the viability of promastigote forms of L. (L). amazonensis and macrophages C57* - The viability assays were performed on both promastigote forms and macrophages cultivated in absence (control) or presence of increasing concentrations of γCdcPLI (0.78 -50 μg/mL) for 24 h. Regarding the control group (untreated group), γCdcPLI was able to reduce 20% and 50% of the parasite’s viability at 25 and 50 µg/mL, respectively (p < 0.05, Fig. 2A). Interestingly, γCdcPLI decreases the viability of macrophages C57 only at 50 µg/mL compared to control group (p < 0.05, Fig. 2B). γCdcPLI at 50 μg/mL reduced the parasite and macrophage viability around 50% and 30%, respectively, showing to be more cytotoxic to promastigote forms when compared to macrophages at the same concentration (p < 0.05, Fig. 2C). Moreover, the half-maximal inhibitory concentration (IC_50_) against *L. (L.) amazonensis* is 48.9 µg/mL, while the half-maximal cytotoxic concentration (CC_50_) for macrophages C57 is higher than 50 µg/mL (highest concentration tested), revealing a SI > 1.0. These results suggest that γCdcPLI had a possible selective action against parasites, as well as an ideal concentration to cause a better effect on promastigote forms of *L. (L.) amazonensis*.

Antileishmanial activity induced by PLA_2_ inhibitors has been described in the literature. Alvarez et al.,^50^ showed the anti-proliferative effect induced by Imidazolidin-2-one derivative compounds against *L.* (*L*) *infantum* promastigotes. The most active derivative Imidazolidin-2-one showed an important activity against the clinically relevant stage of parasites in comparison with Glucantime^®^ and did not induce toxicity on human fibroblast cells. This same study suggested that Imidazolidin-2-one compounds have a direct antiparasitic effect through a perturbation of phospholipid membrane homeostasis and through the inhibition of the parasite protein kinase C (PKC), an important enzyme that regulates early events of the parasite-macrophage interaction process.^50^ Another study demonstrated that bromoenol lactone (an inhibitor of calcium-independent PLA_2_) and methyl arachidonyl fluorophosphonate (a selective and irreversible inhibitor of cytosolic PLA_2_ and calcium-independent PLA_2_) induced cytotoxicity to 50% of promastigote forms of *L. (L.) amazonensis* at 15.1 ± 3.7 μM and 50.5 ± 7.8 μM, respectively. Moreover, these PLA_2_ inhibitors did not induce cytotoxicity to peritoneal macrophages.^21^

*γCdcPLI treatment promoted a cell cycle arrest at G0/G1 phase in promastigote forms of L. (L.) amazonensis* - Our previous results demonstrated that γCdcPLI was able to interfere in parasite’s viability. Thus, we aimed to assess the impact of γCdcPLI treatment on the cell cycle progression of promastigote forms of *L. (L.) amazonensis*. This measurement was carried out indirectly, through the analysis of DNA content by flow cytometry using a DNA intercalant (PI). Of note, the inference of cell cycle phases (G1, S, and G2/M) through DNA content is a standardised approach, commonly used in several cellular models,^41^ ^,^ ^42^ ^,^ ^51^ especially in trypanosomatids.^43^ ^,^ ^44^ ^,^ ^52^

Briefly, promastigotes were treated (or not, in case of control) with 10 and 50 µg/mL of γCdcPLI for 24 h, and the DNA content was analysed by flow cytometry. Our results demonstrated that γCdcPLI at 50 µg/mL arrested the cells at G1 phase of the cell cycle and, as a consequence, there was a decrease in the percentage of cells in the G2/M phases 24 h post-treatment (p < 0.05, Fig. 2D, E). The proportion of cells in G1 phase increased from 50% to 60% and decreased from 20% to 5% in the G2/M phase after treatment with γCdcPLI at 50 µg/mL (p < 0.05, Fig. 2D, E).

One possible explanation for this phenomenon, based in other study,^30^ is that the treatment with 50 µg/mL of γCdcPLI may have caused a variety of reactions that affect the G2 phase, with impaired synthesis of proteins that will be needed for the next phase of the cycle, as well as impairment by the duplication of organelles in mitosis and cytokinesis.^30^ However, we believe that the most parsimonious hypothesis explaining the observed cell cycle arrest in G1 phase is the presence of DNA damage. Possibly, the cells arrested in G0/G1 phase because they were unable to overcome the G1/S checkpoint, which normally occurs when there is something wrong with the cell, predominantly DNA damage. Similar findings have been demonstrated by other natural or synthetic compounds with antileishmanial activities. It was demonstrated that solidagenone, sesamol, betulinic acid, among others, impaired cell cycle progression of *Leishmania* spp. by increasing the number of cells in the G0/G1 phase, as well as decreasing the proportion of promastigotes at the remaining phases (S and G2/M).^53^ ^,^ ^54^ ^,^ ^55^ ^,^ ^56^ ^,^ ^57^ Therefore, we hypothesise that the γCdcPLI treatment may be causing some type of DNA damage, perhaps DNA breaks as a consequence of apoptotic processes, as demonstrated in other cell models,^58^ which could explain the cell cycle arrest in γCdcPLI-treated promastigotes. Another possibility is that treatment with γCdcPLI would impair proliferation,^58^ leading to an increase in quiescent (dormant) cells. Although little studied, this phenomenon has been reported in other trypanosomatids as a result of drug exposure.^59^ ^,^ ^60^ To obtain more evidence to support any of these hypotheses, further experiments need to be thoroughly evaluated in subsequent studies.

**Fig. 3:**
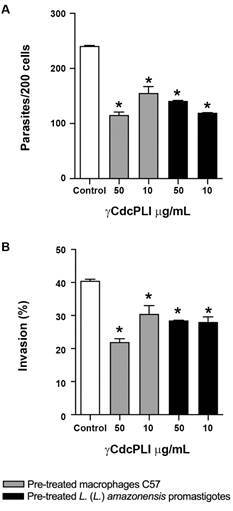
effect of γCdcPLI on the invasion of *Leishmania (Leishmania) amazonensis* promastigotes in macrophages C57. Macrophages C57 or promastigotes were pre-treated with γCdcPLI (10 and 50 µg/mL) prior infection for 24 and 1 h, respectively, and then allowed to interact with macrophages for 4 h. (A) Total number of intracellular parasites, and (B) Percentage of infected cells in a total of 200 cells examined randomly. Data are expressed as mean ± standard deviation (SD). Significant differences were determined using one-way analysis of variance (ANOVA) and Dunnett’s multiple comparisons test. ^*^Statistically significant difference (p < 0.05) compared with the control.

**Fig. 4:**
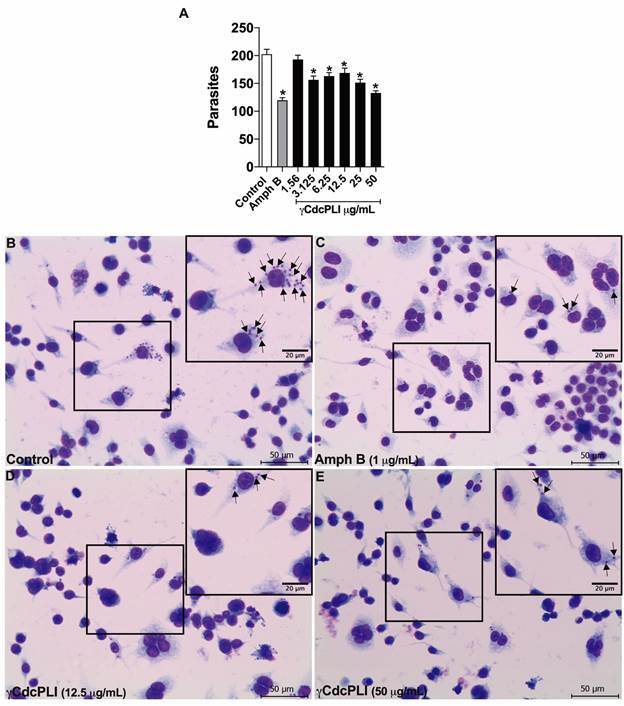
intracellular killing assay. Macrophages C57 were infected with *Leishmania (Leishmania) amazonensis* promastigotes (MOI 10:1) for 4 h, and then treated with twofold serial dilution of γCdcPLI (ranging from 50 and 1.56 µg/mL) or culture medium (control group) for 24 h. Amphotericin B (1 µg/mL) were used as positive control against *L. (L.) amazonensis*. (A) Total number of intracellular amastigotes in a total of 100 infected cells examined randomly after treatments. Representative images of treatments are demonstrated, as follows: (B) control, (C) amphotericin B (1 µg/mL), (D) γCdcPLI (12.5 µg/mL) and (E) γCdcPLI (50 µg/mL). Data are expressed as mean ± standard deviation (SD). Significant differences were determined using one-way analysis of variance (ANOVA) and Dunnett’s multiple comparisons test. ^*^Statistically significant difference (p < 0.05) compared with the control. Black arrows show intracellular amastigotes. Scale bars (bottom right): 20 µm or 50 µm.

*γCdcPLI interferes directly in the infective capacity of promastigote forms and modulates the host cell environment* - To gain insights into the leishmanicidal activity of γCdcPLI, we verified the capacity of this protein to interfere with the parasite invasion process. For this purpose, we assessed whether γCdcPLI would be able to target the parasites and/or the cells, by treating infected macrophages, or pre-treating both the promastigotes and macrophages prior to infection. Our data showed that the pre-treatment of promastigote forms of *L. (L.) amazonensis* with γCdcPLI (10 and 50 μg/mL) for 1 h reduced the number of intracellular parasites, as well as the percentage of infected cells in comparison with the untreated control group (p < 0.05; Fig. 3A, B). Moreover, the previous treatment of macrophages C57 with both concentrations of γCdcPLI also reduced the percentage of parasite-infected macrophages C57 (p < 0.05; Fig. 3B) and consequently decreased the number of invaded parasites relative to the control group (p < 0.05; Fig. 3A). Also, our data revealed that treatment for 24 h with γCdcPLI (50 to 3.125 μg/mL) were able to inhibit the intracellular parasite multiplication compared to the untreated group (p < 0.05; Fig. 4A), thus highlighting the antiparasitic action of γCdcPLI upon amastigotes forms. As expected, the gold standard treatment with amphotericin B (1 μg/mL) controlled the parasite replication within macrophages related to the control group (p < 0.05; Fig. 4A). It was not possible determine the IC_50_ value against intracellular forms of *L. (L.) amazonensis*. Representative images are shown (Fig. 4B-E).

Several prokaryotic and eukaryotic pathogens produce PLA_2_s that not only function as an important virulence factor, but also have been associated with the penetration process, responsible for entering host cells, and many others intracellular mechanisms.^16^ Thardin et al.,^61^ reported the role of parasite and host cell phospholipases in eicosanoid production by mouse peritoneal macrophages during *T. gondii* invasion. The authors showed that the pre-treatment of tachyzoites with PLA_2_ inhibitors (*e.g.*, 4-p-bromophenacyl bromide and quinacrine), in the absence of Ca^2+^, culminated in a reduction of parasite invasion into macrophages. Moreover, the activities of the cyclooxygenase and lipoxygenase pathways were down-modulated when macrophages were pre-treated with these PLA_2_ inhibitors, which led the authors to suggest that the parasites activated the host cell PLA_2_.^61^

*Leishmania* spp. are intracellular protozoans capable of scavenging glycerophospholipids from host cells and degrading them *via* the PLA_2_ activity, which suggests that these parasites can remodel exogenous lipids into their own *via* the Lands cycle.^16^ ^,^ ^62^ ^,^ ^63^ Degrading activities of PLA_2_s were also reported in *Leishmania*, and they could be involved in the biosynthesis of lipophosphoglycan, the main macromolecule on the surface of the procyclic promastigotes.^64^ The modification of phospholipid composition of infected macrophages has been described, with increasing levels of lysophosphatidylcholine, an effect that may reflect, indirectly, the action of endogenous/parasite PLA_2_ on the macrophage.^65^

Passero et al.,^66^ demonstrated that macrophages infected with *L. (L.) amazonensis* treated with PLA_2_ had more intracellular amastigotes relative to the control group. Furthermore, this study showed an association between intracellular parasitism and PGE_2_ production by infected macrophages.

The involvement of PLA_2_ can be considered as an additional mechanism by which *L. (L.) amazonensis* parasites infect, modulate inflammation and persist in the host, suggesting that the inhibition of this molecule can shed light on important targets for the host-parasite interaction.^67^ ^,^ ^68^ ^,^ ^69^ In this way, these findings are in accordance with our work, which can bring new antileishmanial approaches based on the role of PLA_2_s in parasitism.

In summary, our data indicate that γCdcPLI has a direct effect on parasites, since this protein was highly toxic against promastigote forms, culminating in a reduction of the parasite invasion rate. However, even though the mechanism of action of γCdcPLI appears to be specific against the parasites, an additional and non-exclusive hypothesis to explain the impact of this inhibitor against *L. (L.) amazonensis* could involve the modulation of the host cell environment, since the treatment of macrophages prior or after infection also impaired the parasite growth.

In conclusion, the present study demonstrated the antileishmanial effects of γCdcPLI, a PLA_2_ inhibitor isolated from Cdc snake serum, against *L. (L.) amazonensis.* γCdcPLI presented high toxicity against the parasites, inducing cell cycle arrest that is probably a consequence of DNA damage. Furthermore, γCdcPLI treatment interfered with parasite invasion and intracellular proliferation in host cells. Thus, our results provide evidence of the potential use of PLA2 inhibitors as an interesting approach to studying the pathogenesis of infectious diseases, as well as a model for the discovery of relevant targets in parasites and/or host cells, for the design of new compounds against parasitic illnesses.
