# Supplementary material for: Antileishmanial effects of γCdcPLI, a phospholipase A2 inhibitor from Crotalus durissus collilineatus snake serum, on Leishmania (Leishmania) amazonensis
Source: Mem Inst Oswaldo Cruz. 2023 Nov 27;118:e220225. doi: 10.1590/0074-02760220225 (PMC10690931; doi:10.1590/0074-02760220225)
Supplement: Supplementary file 1 [file 1678-8060-mioc-118-e220225-s.pdf]

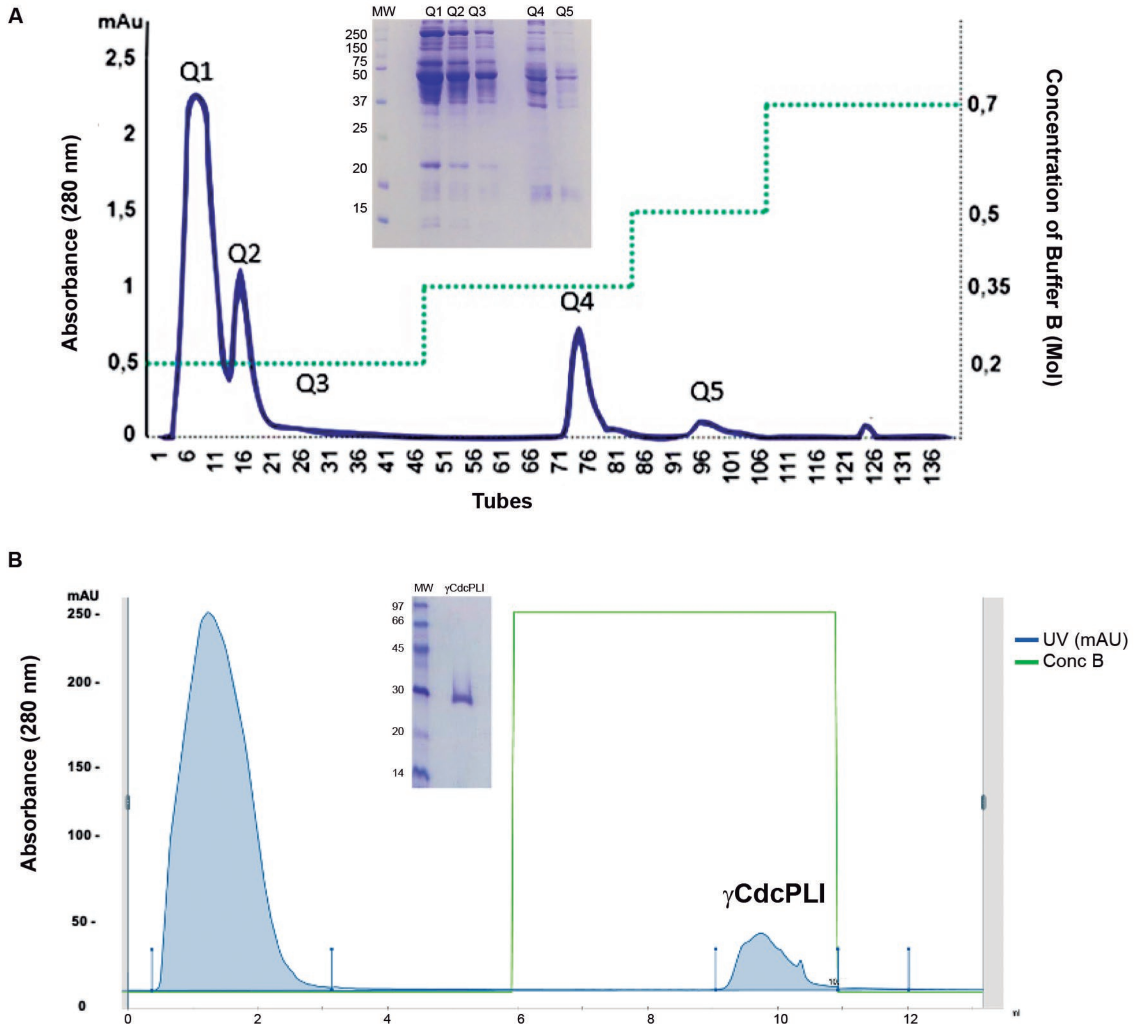

Purification of γCdcPLI inhibitor from C*rotalus durissus collilineatus*' serum. (A) 120 mg of serum was dissolved in 1 mL of 0.05 M sodium phosphate buffer (pH 6.5) containing 0.2 M NaCl, applied to ion exchange chromatography (Q-Sepharose), equilibrated and eluted with 0.05 M sodium phosphate buffer with different NaCl concentrations (0.2 M, 0.35 M, 0.5 M, and 0.7 M) at a flow rate of 12.0 mL/h at 25ºC. (B) Affinity chromatography on NHS-Hitrap (N-hydroxysuccinimide) coupled with BnSP-7 (Buffer A: 10 mM Tris-HCl buffer, pH 7.5; Buffer B: 100 mM glycine-HCl buffer, pH 2.0).
